# Get the Picture? Goodness of Image Organization Contributes to Image Memorability

**DOI:** 10.5334/joc.80

**Published:** 2019-08-12

**Authors:** Lore Goetschalckx, Pieter Moors, Steven Vanmarcke, Johan Wagemans

**Affiliations:** 1 Laboratory of Experimental Psychology, KU Leuven, Tiensestraat, Leuven, BE

**Keywords:** Memory, Visual perception, Vision

## Abstract

According to Gestalt psychologists, goodness is a crucial variable for image organization. We hypothesized that these differences in goodness contribute to variability in image memorability. Building on this, we predicted that two characteristics of good organizations, (i) fast, efficient processing and (ii) robustness against transformations (e.g., shrinking), would be characteristic of memorable images. Two planned (Study 1, Study 2) and one follow-up (Study 3) study were conducted to test this. Study 1 operationalized fast processing as accuracy in a rapid-scene categorization task (“categorizability”). Study 2 operationalized robustness against shrinking as reaction time in a thumbnail search task (“shrinkability”). We used 44 real-life scene images of 14 semantic categories from a previous memorability study. Each image was assigned a categorizability and shrinkability score. The predicted positive relation between categorizability and memorability was not observed in Study 1. A post-hoc explanation attributed this null result to a masking role of image distinctiveness. Furthermore, memorable images were located faster in the thumbnail search task, as predicted, but Study 2 could not rule whether this was merely a result of their distinctiveness. To elucidate these results, Study 3 quantified the images on distinctiveness and statistically controlled for this variable in a reanalysis of Study 1 and Study 2. When distinctiveness was controlled for, categorizability and memorability did show a significant positive correlation. Moreover, the results also argued against the alternative explanation of the results of Study 2. Taken together, the results support the hypothesis that goodness of organization contributes to image memorability.

In our modern, digital world, you never have to look very far to encounter hundreds of photographic images. They are everywhere around us. With their predominance increasing, so seems the interest in understanding their properties. For example, researchers have asked what will make an image popular on social media (e.g., McParlane, Moshfeghi, & Jose, 2014), what will make it aesthetically pleasing (e.g., Kong, Shen, Lin, Mech, & Fowlkes, 2016), or where in the image people tend to fixate (e.g., Judd, Ehinger, Durand, & Torralba, 2009).

One interesting image property that has been gaining attention is image memorability, or the likelihood that an image will later be recognized by observers as having been seen before (Isola, Xiao, Parikh, Torralba, & Oliva, 2014). Traditionally, questions about visual memory had been mostly about its capacity or fidelity. Isola et al. (2014) were the first to comprehensively study interstimulus variability in visual memory performance at the image level. Using a repeat-detection memory task, in which participants watch a sequence of images and press a button whenever they see a repeat of a previously shown image, the researchers quantified 2222 images on memorability by computing the proportion of participants who correctly recognized the image upon its repeat. They found high levels of consistency of the memorability scores across participants. The finding has since been replicated with different image sets, such as other scene images (Bylinskii, Isola, Bainbridge, Torralba, & Oliva, 2015), face images (Bainbridge, Isola, & Oliva, 2013), data visualizations (e.g., charts; Borkin et al., 2013), and a huge image set consisting of images of different kinds (LaMem; Khosla, Raju, Torralba, & Oliva, 2015). Moreover, memorability rankings seem to be stable across image contexts (Bylinskii et al., 2015), across time (Goetschalckx, Moors, & Wagemans, 2017; Isola et al., 2014), and across memory paradigms (Goetschalckx et al., 2017). Together, these findings support the concept of memorability as an intrinsic image property.

Interestingly, image memorability does not seem to simply boil down to image popularity or other image properties. Popular images are not necessarily also memorable (Khosla et al., 2015). In a similar vein, memorability is also separate from interestingness (Isola et al., 2014), aesthetics (Isola et al., 2014; Khosla et al., 2015), or an image’s ability to capture attention or cause priming effects (Bainbridge, 2017). Instead, memorability seems to constitute an image property, the origins of which remain unclear. Nevertheless, studies have pointed towards the existence of distinct memorability neural signatures (Bainbridge, Dilks, & Oliva, 2017; Khaligh-Razavi, Bainbridge, Pantazis, & Oliva, 2016).

The establishment of the concept of memorability as an intrinsic, separate image property, raised the question of how it can be predicted and explained. When it comes to automatically predicting an image’s memorability without the need for human annotations, good results have been achieved using global image descriptors such as GIST (Isola et al., 2014; Oliva & Torralba, 2001). Even better results were obtained with a convolutional neural network (CNN; Khosla et al., 2015). While these techniques are very valuable for automated prediction, they do not readily allow us to understand what exactly makes an image memorable. Although, admittedly, there are some ways of gaining more insight from them. Khosla et al. (2015), for example, revealed that their CNN particularly predicted high memorability scores for close-ups of humans, faces and objects, and also often for images containing animals or text. Among the more human-understandable image features that have been investigated so far, are color features, such as mean hue, mean saturation, etc. They only correlated weakly with memorability (Isola et al., 2014). Similarly, most object statistics, such as object counts and pixel coverage, were not very predictive of memorability either (Isola et al., 2014). However, when the semantic label of the scene and the objects within it was taken into account, a support vector regression trained on labeled object statistics did explain a considerable amount of variability in memorability: ρ = .54. People, interiors, foregrounds, and human-scale objects seemed to contribute positively to memorability, while the opposite was true for exteriors, wide angle vistas, backgrounds and natural scenes (Isola et al., 2014). In a further attempt to better understand memorability, Isola, Xiao, Parikh, Torralba, & Oliva (2011) had Amazon’s Mechanical Turk workers annotate their 2222 images with an extensive list of attributes, such as *lot going on, funny, famous place*, etc. They used a feature selection scheme to identify attributes playing a role in memorability. *Enclosed space, face visible, tells a story*, and *recognize place* were found to be positively related to memorability, whereas *peaceful* had a negative relation.

Despite the invaluable work reviewed above, the factors driving memorability are not yet fully understood to date. There is still a fair amount of variability left unexplained. Previous work has mostly focused on either low-level visual features (e.g., mean overall hue) or more abstract, high-level characteristics (e.g., the type of content), except maybe for some of the object statistics studied by Isola et al. (2014), which could, in a way, be considered to be more mid-level. Here, we aimed to contribute to the understanding of image memorability by focusing more on factors at that intermediate level of the visual hierarchy. More specifically, we hypothesized that part of the variability in image memorability resides in the “goodness” of an image’s organization.

It has long been argued by Gestalt psychologists that visual stimuli differ in how well they can be organized. Good organizations are believed to be characterized by, among other things, regularity, symmetry, and simplicity (Koffka, 1935). In a good organization, the constituting parts are combined into a strong, coherent whole, following Gestalt principles (Wertheimer, 1923/2012; for extensive reviews, see Wagemans, Elder, et al., 2012; Wagemans, Feldman, et al., 2012). For example, a dot lattice is considered to constitute a better organization than a random dot pattern because of its larger degree of regularity, and the set of all possible dot lattices can also be ranked in terms of goodness based on their degree of symmetry (Kubovy, 1994; Kubovy & Wagemans, 1995). In his information theoretic approach, Garner (1962) postulated that good patterns are those that have few equivalents. In the popular game “Tetris”, the O-tetromino would be the one with the highest goodness, as it has no equivalents other than itself (i.e., it does not change under the rotation transformation). The I-tetromino scores only a little less, as it only has two equivalents (landscape and portrait), whereas the T-tetromino has four. Garner and Clement (1963) tested his theory with dot patterns and found that participants assigned higher goodness ratings to those patterns for which an independent group of participants had assigned fewer other patterns to its group of equivalents. Other ways to quantify goodness have been proposed by Hochberg and McAlister (1953) and by van der Helm and Leeuwenberg (1996).

While the aforementioned work dealt with relatively basic, easy to parametrize stimuli (e.g., dot patterns), goodness is much harder to quantify with more rich and complex visual material (e.g., paintings and photographic images). Yet, Gestalt notions similar to goodness have made their way into that literature as well. For example, according to Arnheim (1954/2004) a visual artwork with a good organization is one that is in visual balance. It is believed that visual balance facilitates the combination of pictorial parts into a coherent, comprehensive whole and thus helps to convey the meaning of the visual display. Arnheim identified a structural skeleton consisting of multiple axes (horizontal, vertical, and diagonal) and nine primary locations (including the center of the frame), which are said to attract pictorial parts towards them. A composition is in visual balance when all the attractive forces, as Arnheim calls them, cancel each other out and everything seems at rest (e.g., parts placed at the center or in symmetric positions to the center). This is opposed to a case in which the pull is stronger in one direction and no such equilibrium is established (e.g., parts predominantly in one part of the picture). This idea is somewhat supported by a study by Abeln et al. (2016), who found that when participants have to crop photographs to make them look as nice as possible, they not only tend to go for high overall saliency, but also for a balance in the distribution of the salient regions. The center-of-mean for saliency was generally close to the geometrical center of the frame. In addition, Jahanian, Vishwanathan, and Allebach (2015) started from a large set of aesthetically highly rated photographs and tried to model the probability of a high saliency value at each pixel location using a mixture of Gaussians. Their results show hotspots similar to Arnheim’s nine primary locations, and share other characteristics with the structural skeleton as well. Another, very much related, if not synonymous concept, is that of visual rightness (Carpenter & Graham, 1971; Locher, Stappers, & Overbeeke, 1999), which also encompasses the notion that there is a right way to arrange the parts in order to maximize the impact on the viewer and that artists tend to know how. Locher et al. (1999) stress that it is not so much specific, individual locations that can be “right”, but the entire spatial system of interrelations. In an empirical investigation, Locher (2003) experimentally manipulated artworks to make them less well-organized. He then presented participants with both the original works and the manipulations and had them decide which one was more likely the real one (i.e., the original). When the manipulation saliently disrupted the spatial organization, participants identified the real one more often than expected by chance, leading Locher to conclude that visually right compositions are salient to viewers, even when they lack formal training in the visual arts. Finally, the concept of a good Gestalt has also been picked up outside the field of psychology and has become popular in photography and design textbooks (e.g., Freeman, 2015; Macnab, 2011).

Of specific interest to the hypothesis about image memorability and goodness of organization raised above, is that goodness of organization has been associated with memory benefits. To quote Attneave, “It has been generally held by Gestalt psychologists that ‘good’ figures are remembered more accurately than ‘poor’ ones” (Attneave, 1955, p. 209). Checkosky and Whitlock (1973), for example, found that better dot patterns, as defined in terms of Garner’s (1962) number of equivalent patterns, are recognized more easily in a recognition memory task (see also Garner, 1974). Using different patterns, Attneave (1955) and Schnore and Partington (1967) found similar results for reproduction memory (see also Garner, 1974). More recently, there has also been a lot of research on the benefits of Gestalt organizational cues for visual working memory (Gao, Gao, Tang, Shui, & Shen, 2016; Peterson & Berryhill, 2013; Woodman, Vecera, & Luck, 2003). Furthermore, Brady, Konkle, and Alvarez (2009) demonstrated that statistical regularities in the input arrays were easier to remember in visual short term memory, which could be attributed to a compression advantage at the encoding stage. In a more recent modeling study, Brady and Tenenbaum (2013) formalized the role of perceptual organization in a probabilistic model of visual working memory in which higher-order structure was explicitly incorporated.

In addition to memory benefits, goodness of organization has also been said to be characterized by faster, more efficient processing (e.g., Garner, 1974). For example, when participants need to sort cards displaying one of two alternative dot patterns into two piles, they do so faster for better patterns (Clement & Varnadoe, 1967). Better patterns also yield shorter reaction times in a discrete reaction time task using classification (Garner & Sutliff, 1974) and seem to suffer less from backward masking (Bell & Handel, 1976). In all these cases, the effects were attributed to faster processing of better patterns. Bell and Handel (1976) also predicted, but did not test, that better patterns would therefore suffer less from short stimulus presentations compared to poor patterns. In a speeded classification task, however, Pomerantz (1977) found that good patterns were encoded no faster than poor patterns. He explained the discrepancy with the earlier results by attributing the earlier results to response bias in favor of the good pattern or to intercept effects resulting from decision or response selection rather than encoding as such.

Better forms and patterns are also more robust against transformation. For example, Stadler, Stegagno, and Trombini (1987, as cited in Luccio, 1999) showed that good forms do not transform into poor forms as easily as vice versa in a stroboscopic transformation experiment. Wagemans (1992, 1993) showed that it is easier to match dot patterns and polygons with their affine and perspective transformed counterparts (corresponding to presenting the stimuli on differently oriented planes relative to the viewer) if they contain mirror symmetry than if they do not. A similar advantage was also obtained for dot patterns with other types of regularities (e.g., collinearity, parallelism) compared to patterns that appear more random (Kukkonen, Foster, Wood, Wagemans, & Van Gool, 1996; Wagemans, Van Gool, Lamote, & Foster, 2000). Another transformation to which good Gestalts are considered to be more robust, is reduction in size. In the world of art directors, it is generally believed that if an image does not speak to observers under minified viewing (i.e., reduced to thumbnail size), it will definitely not work in a magazine either (Koenderink, 2015). Koenderink (2015) proposes that those images that do survive “have ‘something’ that other pictures lack. The ‘something’ evidently has to do with the perceptual organization evoked by them. The images have a Gestalt quality that easily survives reduction to postage stamp size” (Koenderink, 2015, p. 908). Interesting in this regard is a study by Suh, Ling, Bederson, and Jacobs (2003), who proposed a more effective way to generate thumbnails of images than mere shrinking. Their algorithm first crops an image and searches for a cropping window that maximizes the saliency of the result and minimizes its size. The saliency is computed based on both low level features and semantic information (i.e., face detection). They found that their method, when compared to mere shrinking, increases participants’ performance in identifying the thumbnail content after a presentation of 2 s and results in faster search times when participants need to find a target thumbnail among distractor thumbnails. Although not directly tested, one could reason that those images that are good from the start, such that the algorithm cannot contribute anything beyond mere shrinking, will yield better identification and search times after shrinking than those that were not as good from the start and could have benefitted from the algorithm.

Based on the literature reviewed above, we operationalized our main hypothesis that image memorability (at least partly) relates to goodness of image organization into two more specific hypotheses that are more directly testable. Specifically, the work presented here is based on the idea that goodness of organization is characterized by faster, more efficient processing and larger robustness against transformation. First, we hypothesized that memorable images would be processed faster (or, more accurately at ultra-rapid stimulus presentation). In Study 1, we tested this hypothesis using a rapid-scene categorization task (for more details, we refer to the introduction of Study 1; Thorpe, Fize, & Marlot, 1996). Our second hypothesis was that memorable images would be more robust against transformation. This hypothesis was addressed in Study 2. More specifically, we studied a shrinking transformation and developed a thumbnail search task to quantify to what extent an image survives shrinking to thumbnail size and can still convey its meaning (for more details, we refer to the introduction of Study 2). We discuss the results of Study 1 and Study 2 together in an interim discussion, before introducing a third study aimed at clarifying some of those results.

## Study 1: Categorizability

In the General Introduction, we proposed that part of the variability in image memorability resides in the goodness of an image’s perceptual organization. Fast, efficient processing is a characteristic often ascribed to visual displays of good organization and therefore, we hypothesized that memorable images would be processed faster (or, more accurately at ultra-rapid stimulus presentation). In Study 1, we tested this hypothesis adopting a rapid-scene categorization task (Thorpe et al., 1996). On each trial, participants were very briefly (33 ms) presented with a scene image, followed by a mask and then a label. They had to judge whether the label matched the scene or not. We asked two questions. First, we asked whether there was consistent interstimulus variability in this task, as was observed for memorability, in the sense that images would differ consistently in their probability of being categorized correctly at this very short stimulus duration. We will refer to this probability as “categorizability”. Second, we asked whether categorizability correlates positively with memorability. To this end, we selected scene images from a set for which memorability scores were already available (Bylinskii et al., 2015).

### Methods

#### Participants

A total of 147 undergraduate psychology students participated in this study in exchange for course credits (125 women, 22 men).^1^ Ages ranged from 17 to 29 years old (*M* = 18.45, *SD* = 1.37). The study was approved by the Ethical Committee of the Division of Humanities and Social Sciences, KU Leuven, Belgium. All participants gave written informed consent prior to the start of the study.

#### Stimuli

##### Images

**Figure 1:**
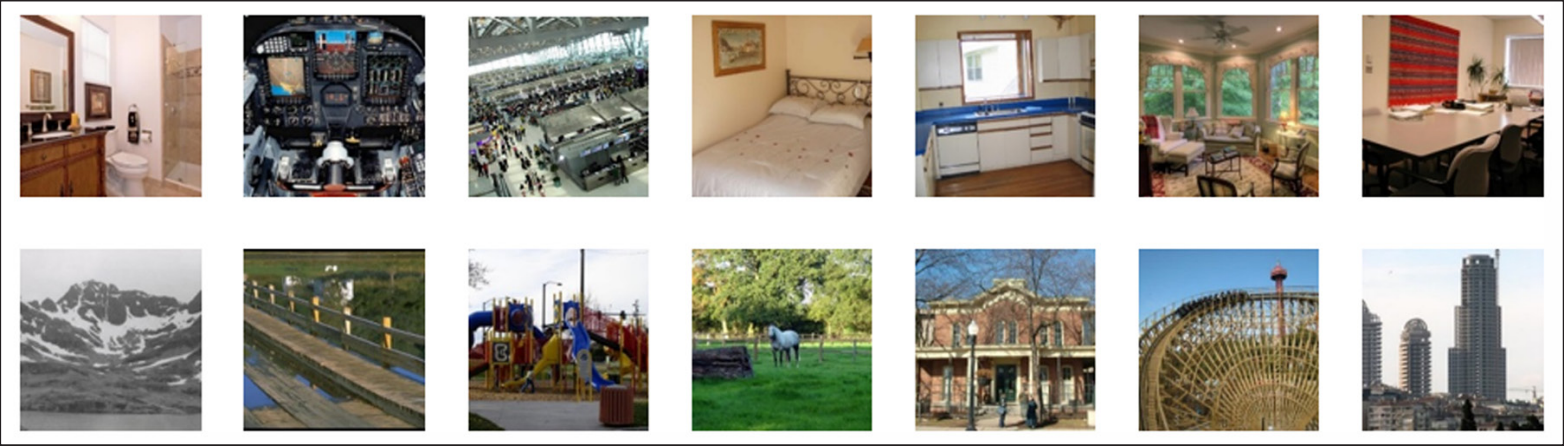
Examples of the selected images. The top row presents an example image for each of the indoor categories. From left to right: *bathroom (badkamer), cockpit (cockpit), airport terminal (luchthavenhal), bedroom (slaapkamer), kitchen (keuken), living room (living)*, and *conference room (vergaderruimte)*. The bottom row presents an example image for each of the outdoor categories. From left to right: *mountain (berg), bridge (brug), playground (speeltuin), pasture (wei), house (gevel van een huis), amusement park (pretpark)*, and *skyscraper (wolkenkrabber)*. The Dutch labels are between brackets. All images were taken from the FIGRIM-dataset (Bylinskii et al., 2015).

We selected 14 of the 21 scene categories in the FIGRIM-dataset (Bylinskii et al., 2015), in such a way that the final selection contained equal numbers of indoor and outdoor categories (see Figure 1). We specifically avoided including image categories that are too similar (e.g., *pasture* and *golf course*). The original English category labels were translated to Dutch. All but one category received a Dutch label that paralleled the English label. The only exception was the scene category *house*, which was more loosely translated as *gevel van een huis* (façade of a house). This was to avoid confusion with certain indoor categories (e.g., *bedroom*, which is part of a house). For each category, we then randomly selected 44 images after excluding those we deemed unsuitable for the current purposes. Reasons for exclusion were: not having been scored on memorability, having an ambiguous category membership (e.g., an image of a pasture on a mountain could belong to either *pasture* or *mountain*), and containing text that is not part of the scene (e.g., the date the image was captured). An additional two images were selected for each category: one of these was used for the familiarization procedure (i.e., example images), the other for the practice trials (see Procedure). All the above selections were carried out once, such that all participants received the same stimulus set with the same assignment to familiarization phase, practice phase, and main task. The IDs of the selected images, together with the category label (both original and translated) are available for download (see the Data Accessibility Statement).

##### Masks

With the exception of the example images, we generated a colored mask for each of the selected images. This was achieved by adding random deviations to the phase spectrum of each image in the Fourier domain, while preserving the original amplitude spectrum (Hansen & Loschky, 2013; Vanmarcke, Noens, Steyaert, & Wagemans, 2017). For each given point of an image, the random deviation was constant across the three color dimensions (RGB).

#### Task and Procedure

Participants were invited to a computer lab of the university, where they were seated individually in front of a computer. All computers had a 21.5-inch TFT-monitor with a resolution of 1920 × 1080 px and a refresh rate of 60 Hz. The study started out with a short familiarization procedure. During this procedure, an example image of each category was shown on the screen, along with the corresponding category label. The presentation was self-paced and in a random order. The familiarization procedure was intended to give participants an idea of which scene categories would be involved in the actual rapid-scene categorization task.

**Figure 2:**
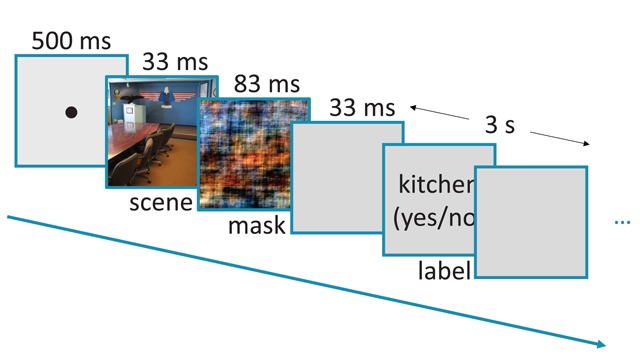
Schematic of the rapid-scene categorization task.

Each trial of this rapid-scene categorization task (see Figure 2) consisted of the consecutive presentations of a fixation dot (500 ms), the target image (33 ms) and its corresponding mask (83 ms). Both the target image and the mask were presented at a size of 512 × 512 px. The mask was followed by a brief interval of 33 ms in which only the grey background was presented. Finally, a scene label appeared on the screen. The instructions were to indicate whether the label matched the scene, ‘yes’ (J-key) or ‘no’ (F-key). There was a response limit of 3 s. Upon the registration of a response, the label disappeared and the screen remained blank for the remainder of the 3-s response interval (before the onset of the next experimental trial). When participants failed to respond within this 3-s response interval, the response was regarded as incorrect and the next trial was initiated. Importantly, a random half of the target images of a given category were presented with the congruent category label (e.g., a random half of the bathroom images were followed by the label ‘Bathroom?’). The remaining half of the target images were presented with an incongruent category label (e.g., images of a bathroom followed by the label ‘Kitchen?’). The incongruent labels were randomly selected from the same superordinate category (i.e., indoor or outdoor). This resulted in a total of 616 trials per participant, of which 308 (22 × 14) were congruent (requiring a ‘yes’ response of the participant) and 308 (22 × 14) were incongruent (requiring a ‘no’ response of the participant). The 14 target categories were presented randomly across trials. The order of the trials was also fully randomized over categories. All the aforementioned randomizations were performed separately for each participant.

Fourteen practice trials, presenting the 14 practice images (see Stimuli), were added to the beginning of the task to acquaint participants with the task (especially the short presentation durations). During these practice trials, but not in the main experiment, feedback was provided. In total, the task lasted about an hour. Participants were offered 11 self-timed breaks, one immediately after the practice trials, the others every 56 trials.

#### Categorizability Scores

To quantify how easy it is to categorize a certain image rapidly, we assigned each image a “categorizability” score. These scores were computed using a similar method as the one used by Bylinskii et al. (2015) to compute the memorability scores for the images we used here (also see Isola et al., 2014). Specifically, for each image we calculated the proportion of participants who correctly categorized the image on the congruent trials. As a result, the scores were based on an average of 74 responses per image.

### Results

Whenever we describe hypothesis tests in this section or any of the other Results sections, the adopted alpha level was .05 unless otherwise indicated.

#### General Performance

Despite the brief and masked target scene presentation, participants were able to categorize the images relatively well, with a mean percentage correct of 78% (*SD* = 8%) and a mean *d’* of 1.69 (*SD* = 0.55; see Macmillan & Creelman, 2005, for an explanation of the *d’*-measure from signal detection theory). This is in line with previous findings on the time-course of masked rapid-scene categorization, showing that remarkably short presentation durations often suffice for observers to be able to extract the gist of a scene (e.g., Fei-Fei, Iyer, Koch, & Perona, 2007; Thorpe et al., 1996; Vanmarcke & Wagemans, 2015). Average percent correct scores were higher on incongruent trials (*M* = 84%, *SD* = 10%) than on congruent trials (*M* = 73%, *SD* = 13%); *t*(146) = 8.75, *p* < .001. This is likely due to an overall bias to respond ‘no’ when being uncertain about the correct response (mean β = 1.52; *SD* = 0.59; see Macmillan & Creelman, 2005). When unsure, participants perhaps did not feel inclined to respond ‘yes’ knowing there were 14 categories involved in the task and only one would imply a match. They might not have picked up on the fact that there were as many congruent as incongruent trials.

#### Categorizability Scores

Table 1 shows descriptive statistics for the categorizability scores per category. There is considerable variation across the categories in the mean categorizability scores, suggesting that some categories were easier than others in this rapid-scene categorization task. Notice that the easiest categories (highest mean categorizability score) also showed the least variation in the categorizability scores of their members. This was confirmed when correlating the mean and the standard deviation of the categorizability scores of a category: *r*(12) = –.81, *p* < .001. This is probably due to a performance ceiling effect (leading all participants to perform more similar on the easier task categories).

**Table 1:** Descriptive Statistics for Categorizability Scores per Category.

	Living room	Bridge	Kitchen	Bathroom	Conference room	Bedroom	Airport terminal	Amusement park	Playground	Mountain	Cockpit	House	Pasture	Skyscraper

Mean	.55	.60	.63	.64	.65	.66	.67	.70	.73	.82	.85	.87	.88	.89
Median	.56	.64	.65	.69	.64	.69	.69	.76	.79	.85	.88	.89	.90	.90
SD	.17	.21	.15	.19	.18	.16	.14	.23	.19	.09	.10	.08	.10	.06
Min	.22	.17	.20	.27	.30	.28	.27	.15	.23	.61	.43	.61	.40	.77
Max	.87	.97	.95	.92	.92	.94	.90	.96	.94	.97	.97	.98	.99	.97

##### Consistency across participants

**Figure 3:**
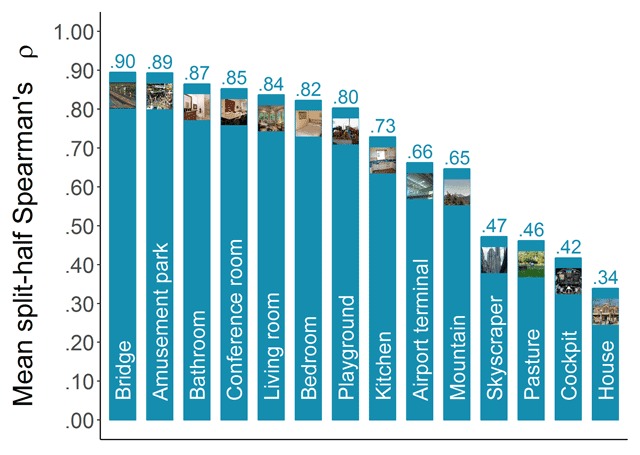
Consistency of categorizability scores across participants. Mean-split-half Spearman’s rank correlations were calculated based on 1000 random splits.

To assess the consistency of the categorizability scores across participants, we applied the same method that has previously been applied to memorability scores (e.g., Bylinskii et al., 2015; Goetschalckx et al., 2017; Isola et al., 2014). That is, we randomly split our participant pool into two halves and calculated Spearman’s rank correlation (ρ) between the categorizability scores based on the responses of the first half and those based on the responses of the second half. Repeating this 1000 times and averaging the resulting correlations provided an estimate of the consistency. Figure 3 presents the mean split-half Spearman’s rank correlations in function of the category. Most categories reach high levels of consistency (ρ’s up to .90), suggesting that categorizability can also be considered a meaningful image property. However, some categories seem to be lagging behind and show lower levels of consistency with the available number of responses. Suspecting this might be due to range restriction for the easier categories, we tested the Pearson (*r*) correlation of the consistency estimate with the mean categorizability for a category, as well as with the standard deviation of the categorizability scores. The results were *r*(12) = –.87, *p* < .001, and *r*(12) = .91, *p* < .001, supporting our suspicion that these low reliability scores were due to high mean categorizability scores.

##### Categorizability versus memorability

**Figure 4:**
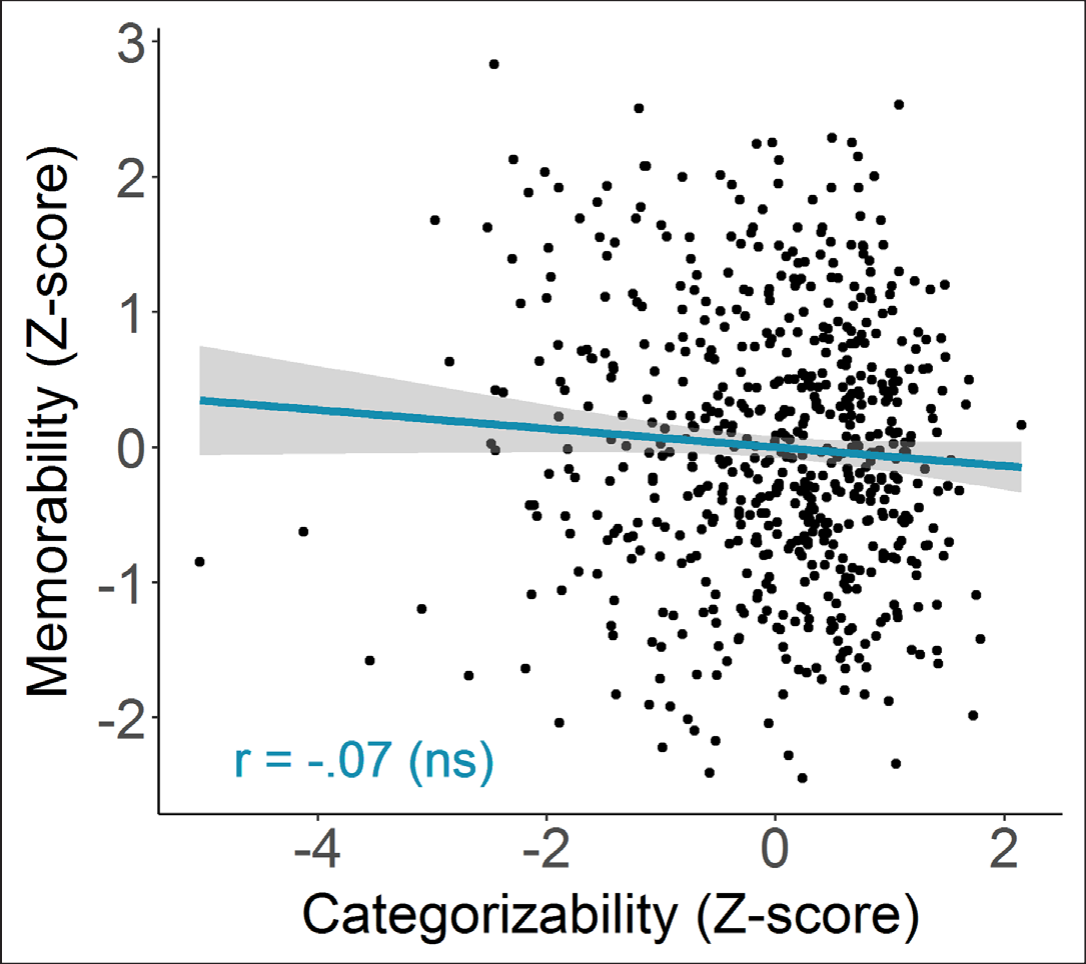
Memorability in function of categorizability. Each point represents an image (N = 616). The blue line indicates the best fitting regression line and the bands show 95% confidence intervals. The corresponding Pearson correlation is indicated in the bottom left corner.

Figure 4 shows a scatterplot of the *z* score of the memorability score (taken from Bylinskii et al., 2015; AMT1: within-category experiment) for each image against the *z* score of its categorizability score (collected here). *Z* scores were computed per category in order to ensure that any general correlation between categorizability and memorability we might observe would not be distorted by differences in mean categorizability and memorability across categories (i.e., to ensure that this correlation would not be driven by between-category differences rather than within-category differences). A one-tailed hypothesis test for the Pearson correlation (*r*) between the two *z*-scored variables failed to reject the null hypothesis that the correlation was smaller than or equal to zero : *r*(614) = –.07, *p* = .96. If anything, the nominal correlation value was negative. We offer a possible explanation for this result in the Interim Discussion following Study 2.

## Study 2: Shrinkability

While in Study 1, we approached our main hypothesis about image memorability and goodness of perceptual organization from the angle of fast and efficient processing, we turned to a different characteristic of good organizations in Study 2. Here, we focused on robustness against transformation and more specifically on a shrinking transformation. We used the same images as in Study 1 and quantified to what extent they survive shrinking to thumbnail size using what we called a thumbnail search task. In this task, participants had to look for a thumbnail version of regular-sized image among eight distractor thumbnails. The rationale here was that it would be easier to find the matching thumbnail for those images that succeed better at conveying their meaning under minified viewing. Therefore, we operationalized the “shrinkability” of an image as being negatively related to the mean reaction time across participants. Analogously to Study 1, we asked two questions: (1) are shrinkability scores consistent across participants, and (2) does shrinkability correlate positively with memorability?

### Methods

#### Participants

A total of 75undergraduate psychology students participated in this study in exchange for course credits (72 women, 3 men).^2^ Ages ranged from 17 to 20 years old (*M* = 18.17, *SD* = 0.62). There was no overlap between the participant pool for this study and Study 1. The study was approved by the Ethical Committee of the Division of Humanities and Social Sciences, KU Leuven, Belgium. All participants gave written informed consent prior to the start of the study.

#### Stimuli

The image set of the shrinkability study was the same as that of the rapid-scene categorization study (Study 1). However, to limit task duration, each participant performed the thumbnail search task for a random selection of 30 out of 44 images per category. This selection process (30 out of 44 images per category) differed for each participant (in a random fashion), providing us with shrinkability scores on all 616 images when collapsing the data of all participants together. The thumbnail images making up the search display of the thumbnail search task were shrunken versions of images selected from the total image set of 44 images per category. The images to be searched for in the practice trials, were the same as those used in the practice trials of Study 1. We did not use example images in this study.

#### Task and Procedure

As in Study 1, participants were invited to a computer lab of the university, where they were seated individually in front of a computer. All computers had a 21.5-inch TFT-monitor with a resolution of 1920 × 1080 px and a refresh rate of 60 Hz.

**Figure 5:**
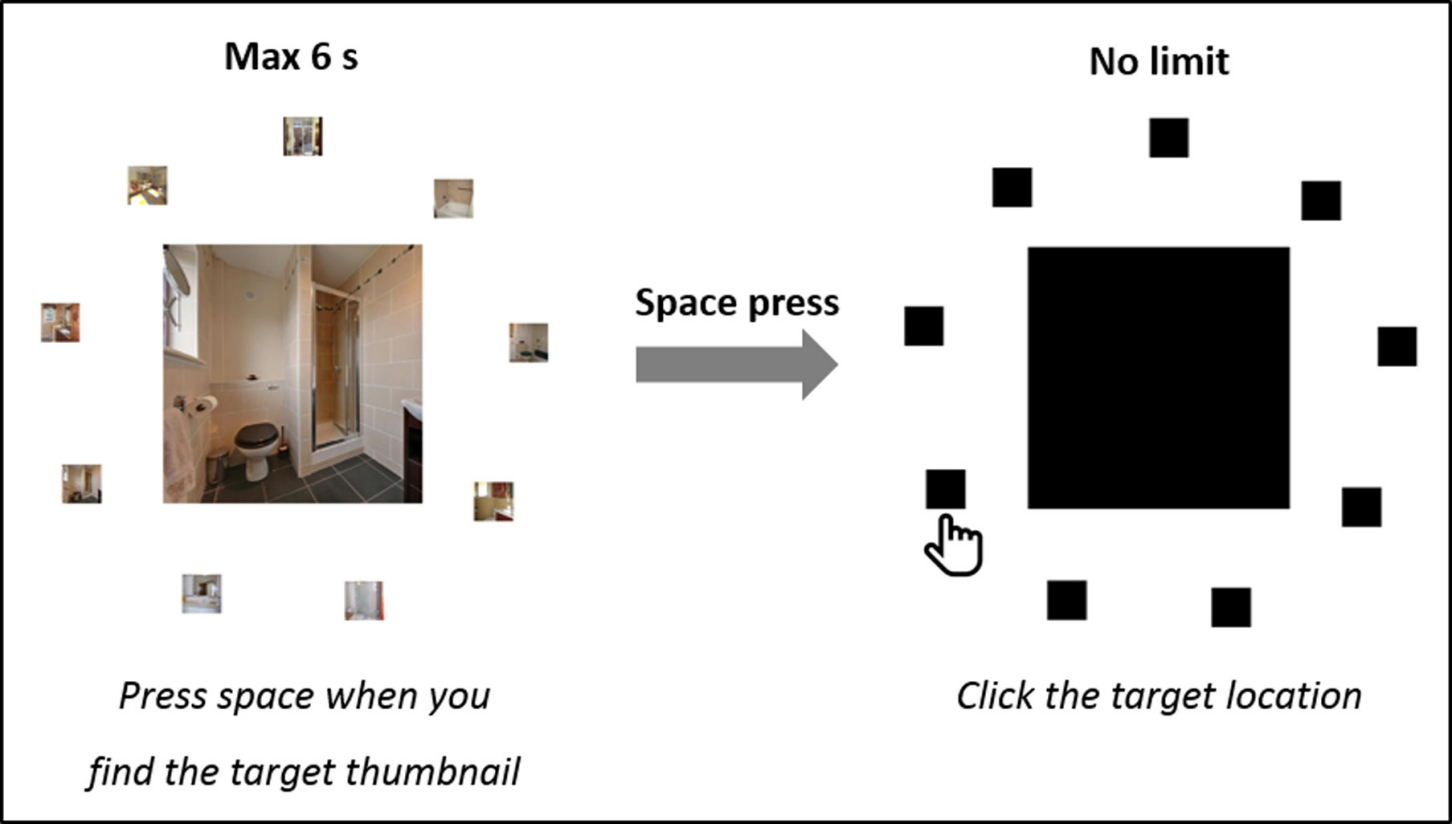
Schematic of the thumbnail search task.

On each trial of the thumbnail search task (see Figure 5), participants were presented with a fixation cross for 500 ms, followed by a circular search display. The search display consisted of a regular-sized image (512 × 512 px; same as in the memory task of Bylinskii et al., 2015) in the center of the screen, surrounded by nine thumbnail-sized images (77 × 77 px) of the same scene category. The thumbnails were centered on an imaginary circle surrounding the regular-sized image in the middle (radius = 470 px). These thumbnails were positioned at equal distances from each other. Furthermore, on each trial, one of the thumbnails was identical (except for the difference in size) to the regular-sized image in the middle. This was the target thumbnail and its location was determined randomly. The eight other thumbnails were distractors and were the shrunken versions of randomly selected images of the same semantic category as the target, distributed randomly over the eight remaining locations. These randomizations were performed separately for each participant.

Participants were instructed to locate the target thumbnail as fast as possible and to press the space bar when they did. As soon as they responded, their reaction time was registered and all images were replaced by black place holders, respecting the size and locations of the original images. Participants were then required to click the place holder that corresponded to the target location. When participants did not respond within 6 s after trial onset, the program immediately moved on to the next trial, thereby skipping the clicking part. Participants were discouraged to guess if they could not find the target thumbnail in time. Instead, they were told not to guess and to let the trial pass by, rather than to risk clicking the wrong location.

There were 420 trials in total (30 images randomly selected from each of 14 scene categories). For each participant, a new random selection from each scene category was created. Furthermore, the order of trials was fully randomized for each participant. Fourteen practice trials, involving the 14 practice images (see Stimuli) as targets, were added to the beginning of the task to help participants adjust to the thumbnail search task. Feedback was provided in the practice trials, but not in the main experiment. In total, the task lasted about an hour. Participants were offered 14 self-timed breaks, one immediately after the practice trials, the others after every 30 trials.

#### Shrinkability Scores

To quantify to what extent a certain image survives shrinking to thumbnail size, we assigned each image a “shrinkability” score. These scores were based on how fast the image could be located when it was presented as a target in the thumbnail search task. That is, for each image we calculated the mean RT across participants, thereby excluding incorrect trials and fixing RTs for trials without a response at 6 s (i.e., the response limit). On average, the scores were based on a total of about 51 RT scores (before exclusion of RTs for incorrect responses). Note that different participants had to look for the target among different sets of distractor thumbnails (i.e., the eight distractors were picked randomly for each participant) and thus the scores also make abstraction of the specific distractors that were present. Also note that, since the scores are RT-based, lower values in fact indicate higher shrinkability. With this operationalization, our prediction translates to a negative correlation between the measure for memorability and the measure for shrinkability, although the predicted relation between the underlying constructs was positive.

### Results

#### General Performance

On average, participants correctly identified the target thumbnail in time on 95% (*SD* = 3%) of the trials and clicked the wrong location on 3% of the trials (*SD* = 3%). The remaining 2% (*SD* = 2%) of the trials were trials where the participant did not find the target thumbnail in time. As mentioned above, RTs for incorrect trials were excluded from further analyses and RTs for no-response trials were set at 6 s. When first averaging across trials and then across all participants, the resulting mean reaction time (RT) amounted to 2.31 s. The fastest participant had a mean correct RT of 1.57 s, the slowest had a mean correct RT of 3.00 s. The participant showing the highest variability in their correct RT, had an *SD* of 1.50 s, the one showing the least had an *SD* of 0.83 s. The average *SD* across participants equaled 1.16 s.

#### Shrinkability Scores

The mean exclusion rate per image amounted to 3%, meaning that an image was incorrectly identified (i.e., participant clicked a distractor thumbnail instead of the target thumbnail) by, on average, 3% of the participants. Table 2 shows descriptive statistics for the shrinkability scores per category. Here too, there seems to be some variation across categories in terms of the mean shrinkability of their respective images. For instance, *skyscraper* images were located fastest, while *playground* images were located most slowly by our participants. Importantly, there is also considerable variation between the shrinkability scores within a category. For example, the most shrinkable *skyscraper* image took less than a second to locate, on average, while the least shrinkable *skyscraper* image took over 3 s. In the next paragraph, we assess the consistency of this inter-item variability across participants.

**Table 2:** Descriptive Statistics for Shrinkability Scores per Category.

	Skyscraper	Bridge	Bedroom	Mountain	Conference room	Amusement park	Pasture	House	Bathroom	Cockpit	Living room	Kitchen	Airport	Playground

Mean	2.03	2.11	2.12	2.21	2.23	2.30	2.30	2.34	2.38	2.45	2.45	2.48	2.50	2.59
Median	2.03	2.05	2.05	2.10	2.25	2.20	2.17	2.29	2.33	2.59	2.49	2.46	2.39	2.59
SD	0.52	0.56	0.40	0.47	0.45	0.71	0.55	0.45	0.47	0.56	0.50	0.44	0.63	0.65
Min	0.88	1.00	1.47	1.18	1.30	1.12	1.46	1.35	1.48	1.05	1.41	1.56	0.89	1.37
Max	3.37	3.69	3.06	3.08	3.20	4.82	3.61	3.38	3.35	3.35	3.39	3.44	4.06	4.14

##### Consistency across participants

**Figure 6:**
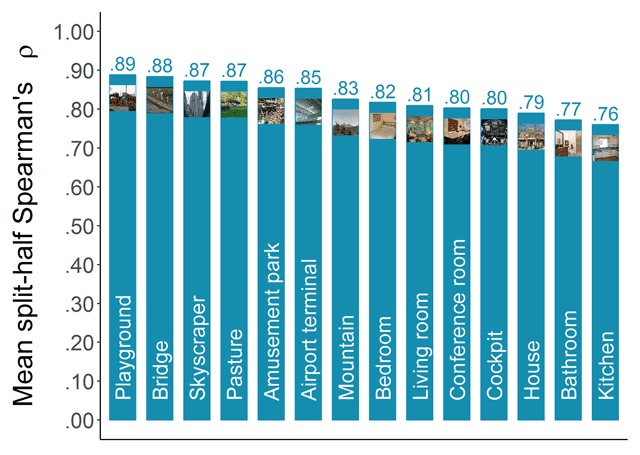
Consistency of shrinkability scores across participants. Mean-split-half Spearman’s rank correlations were calculated based on 1000 random splits.

To assess the consistency of the shrinkability scores across participants, we applied the same method as for the categorizability scores (see above; also see Bylinskii et al., 2015). Figure 6 presents the resulting mean split-half Spearman’s rank correlations in function of category. All categories reach remarkably high levels of consistency with the available number of responses (ρ’s ranging from .76 up to .89).

##### Shrinkability versus memorability

**Figure 7:**
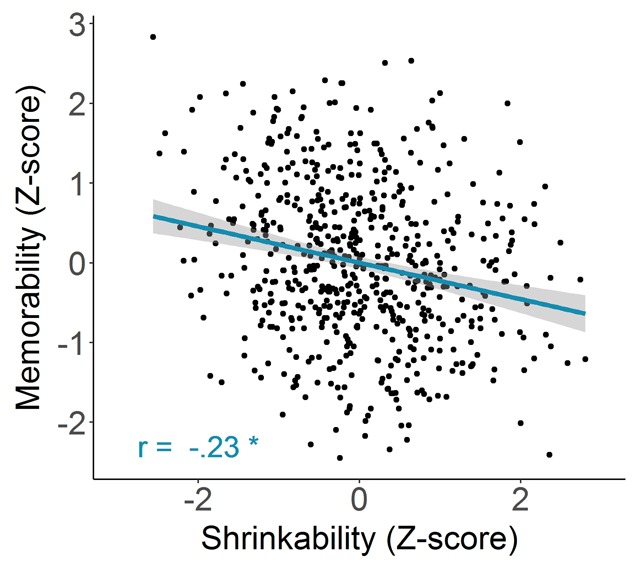
Memorability in function of shrinkability. Each point represents an image (N = 616). The blue line indicates the best fitting regression line, while the bands refer to 95% confidence intervals. The corresponding Pearson correlation is indicated in the bottom left corner. Note that because of the way shrinkability was operationalized (i.e., in terms of RTs), lower values indicate higher shrinkability.

Figure 7 shows a scatterplot of the *z* score of the memorability score (taken from Bylinskii et al., 2015; AMT1: within-category experiment) for each image against the *z* score of its shrinkability score (collected here). As in the analysis of the categorizability scores, *z* scores were computed per category for the shrinkability scores. A one-tailed hypothesis test for the Pearson correlation (*r*) between the measures for shrinkability and memorability rejected the null hypothesis that the correlation was greater than or equal to zero: *r*(614) = –.23, *p* < .001, indicating that more memorable images were located faster in the thumbnail search task.

## Interim Discussion

Using different approaches, both Study 1 and Study 2 aimed to evaluate the main hypothesis, outlined in the General Introduction, that image memorability (at least partly) depends on goodness of image organization.

Study 1 exploited the notion that good organizations are characterized by fast, efficient processing and translated the main hypothesis into the more directly testable hypothesis that memorable images would be processed more efficiently. To test this, Study 1 investigated interstimulus variability in a yes/no rapid-scene categorization task. More specifically, 616 scene images of 14 different categories were assigned a categorizability score based on the proportion of participants who correctly categorized the image in congruent trials (i.e., label matched the scene). The rationale here was that high categorizability scores reflect high processing efficiency. We asked whether categorizability scores are consistent across participants and whether they correlate positively with memorability scores.

Apart from efficient processing, goodness of organization has also been associated with robustness against transformations, such as a shrinking transformation. Based on this idea, Study 2 translated the main hypothesis into the more directly testable hypothesis that memorable images would survive shrinking better. That is, they can still readily convey their meaning under minified viewing. To quantify an image’s shrinkability, we developed a thumbnail search task. The same images used in Study 1 now received a shrinkability score computed as the mean thumbnail search time across participants. The rationale here was that those target thumbnails that survived the shrinking better and still succeeded to convey their meaning, would be located faster. Therefore, lower shrinkability scores in fact represent higher shrinkability, leading us to ask whether shrinkability scores correlate negatively with memorability scores. As in Study 1, we first also assessed the consistency of the scores across participants. Note that as memorability scores were already available from Bylinskii et al. (2015), we did not collect memorability scores in any of the current studies.

In general, the consistency across participants was high, both for categorizability and shrinkability scores (for more discussion, see General Discussion). In the remainder of this section, we focus on the predicted correlations with memorability scores.

**Figure 8:**
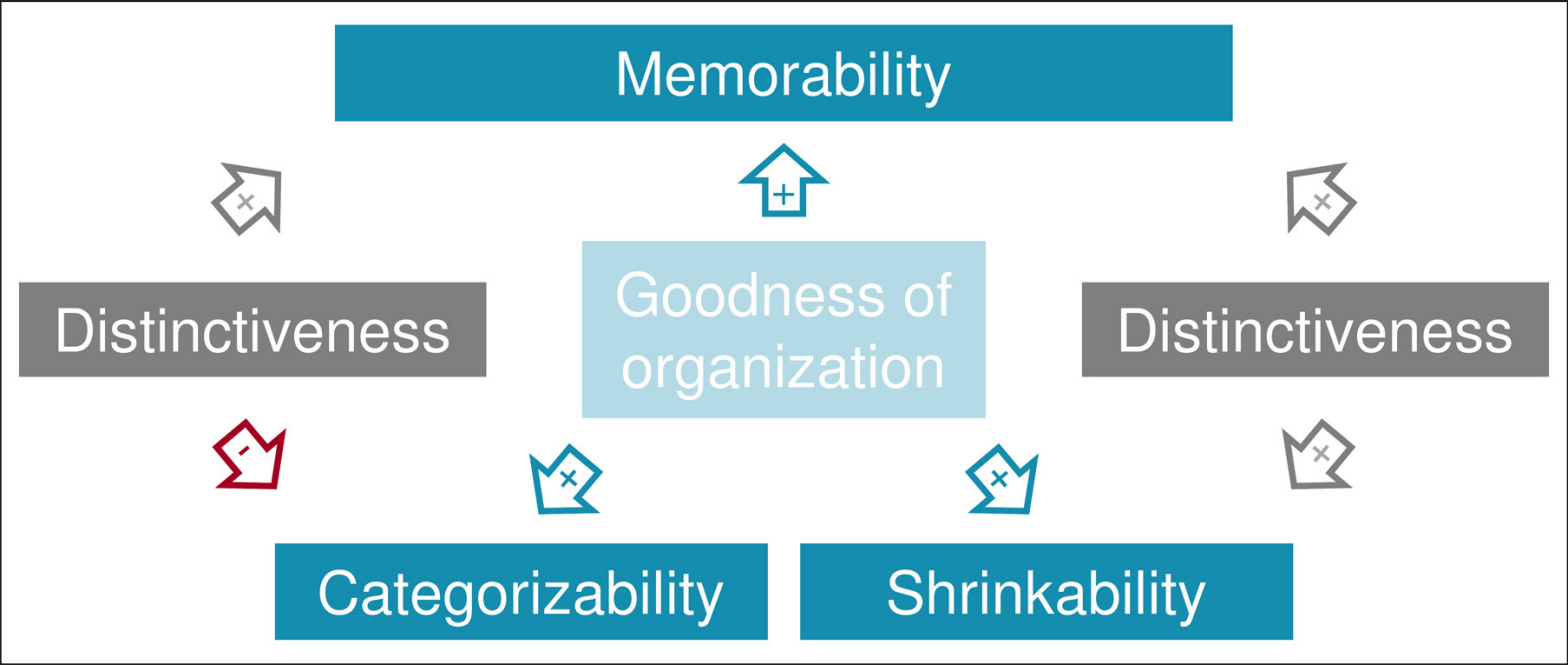
Visualization of the theoretical rationale behind Study 3.

Are memorable images easier to categorize rapidly? The results of Study 1 failed to support this prediction. Initially, we reasoned that memorable images can be processed faster and more efficiently, and that this would result in a higher categorizability score. However, there could be additional factors affecting the categorizability score. It seems reasonable to assume that images that are less typical of their category, would be harder to categorize rapidly. At the same time, atypical images will stand out more from their context or, in other words, be more contextually distinctive. Distinctiveness has long been known to influence memory performance (Bylinskii et al., 2015; Hunt & Worthen, 2006; Konkle, Brady, Alvarez, & Oliva, 2010a; Standing, 1973) and has already been found to correlate positively with memorability (Bylinskii et al., 2015). Thus, if both the fast and efficient processing (associated with goodness) and distinctiveness affect categorizability, while having opposite predicted effects on memorability, then the latter may have masked the effect of the first. This could then explain the null result. The reasoning is visualized in the left-hand side of Figure 8.

A second question was whether memorable images survive shrinking better. The results of Study 2 offered support for this prediction. Shrinkability scores showed a significant negative correlation with memorability scores. That is, memorable images were located faster in the thumbnail search task. We reasoned that this is because memorable images tend to have better organizations and are therefore more robust against the shrinking transformation. However, there is a possible alternative explanation for memorable images yielding shorter reaction times in the thumbnail search task, which is visualized in the right-hand side of Figure 8. Perhaps the result is driven by the fact that memorable images tend to be more distinctive and therefore stand out more from the distractor thumbnails, making it easier to find them. As mentioned previously, distinctiveness and memorability are positively related.

From the above, it is clear that taking the distinctiveness variable into account, is important for understanding the results of Study 1 and Study 2. In order to evaluate the post-hoc explanation for the null result in Study 1 and to rule out the alternative explanation for the result of Study 2, we conducted a third study. In Study 3, we collected distinctiveness scores for the images and reanalyzed the data of Study 1 and Study 2, this time statistically controlling for distinctiveness. Providing a preview of the results, we found that when distinctiveness is controlled for, the results of Study 1 changed in line with our post-hoc explanation and that memorability and categorizability did show a (weak) positive correlation. Furthermore, controlling for distinctiveness did not explain away the correlation between memorability and shrikability observed in Study 2.

## Study 3: Distinctiveness

In Study 3, we aimed to clarify the results of Study 1 and Study 2 and collected distinctiveness scores for the images used there. Distinctiveness is often defined intuitively as the extent to which a stimulus stands out from other stimuli in its context and its relation to memory performance is well established (Bylinskii et al., 2015; Hunt & Worthen, 2006; Konkle et al., 2010a; Standing, 1973). Distinctive stimuli are those that are odd, unique, unexpected, or atypical in a given context. Those last four words are critical, because a stimulus can be all of those things in one context, but not in another. This point is sometimes made explicit by referring to distinctiveness as contextual distinctiveness (e.g., Bylinskii et al., 2015). The effects of distinctiveness on, for example, memory were traditionally often examined by designing the context in a certain way. In the isolation paradigm (e.g., von Restorff, 1933), for example, participants study a collection of items, of which a small minority differs from the majority on some feature. The minority items are generally remembered better. Furthermore, Konkle et al. (2010a) studied distinctiveness in a visual memory task and found that memory for object images decreases with the proportion of exemplar images of the same object category presented in the study phase. A similar result was found for scene images (Konkle, Brady, Alvarez, & Oliva, 2010b), although overall performance was still high in both stimulus categories.

In the current work, we did not seek to manipulate the context. Instead, the context was given (i.e., the images used in Study 1 and Study 2) and the goal was to quantify each image’s distinctiveness with respect to this context. Directly quantifying distinctiveness is somewhat challenging. Murdock (1960) proposed a method that is based on the sum of differences on a certain dimension between a stimulus and all other stimuli in its context. Except, in his study there was only a single dimension at play (i.e., tone intensity) and the dimension was known. For rich scene images, it is less obvious which dimensions need to be evaluated. In this regard, Lukavskỳ and Děchtěrenko’s (2017) sparseness measure comes to mind, which could be interpreted as a distinctiveness measure for images. They represented each of their images using deep features extracted from a pretrained CNN and calculated a sparseness score as the distance of an image to its n^th^ nearest neighbor. The distances were in turn calculated as the Euclidian distances between the CNN-feature vectors. Image sparseness was significantly positively related to recognition sensitivity. In a similar vein, Bylinskii et al. (2015) also represented their images using CNN-features and quantified the distinctiveness as the negative log likelihood under the probability distribution over the images (or better, their CNN-feature vectors) in the context. The resulting distinctiveness scores were significantly positively correlated with the memorability scores. In both studies, the CNN was pretrained to perform a classification task and the features were extracted from the final layer before the output layer yielding the (semantic) class predictions. Hence, the derived measures were likely to capture a rather conceptual, semantic type of distinctiveness.

An advantage of the computer-based measures discussed above is that they can be computed automatically. On the other hand, it is uncertain to what extent they truly capture what is distinctive to humans. A different approach is to have participants rate the distinctiveness of an image. This approach has often been used in research on face distinctiveness and memory, where the correlation has also been observed (e.g., Valentine, 1991; Wickham, Morris, & Fritz, 2000). This is the approach we adopted here. We collected distinctiveness scores in an online study where participants were first familiarized with the image context, and then provided distinctiveness ratings on a 7-point scale. Participants were not stimulated to rely mainly on conceptual, semantic features. Instead, the instructions were kept very open and general. Henceforth, we will refer to this measure as perceived distinctiveness. To assess whether our perceived distinctiveness measures tapped into the same source of variability as the measures derived from CNNs, we compared our perceived distinctiveness measure with existing computer-based measures (Bylinskii et al., 2015, hereafter CNN-likelihood; Lukavskỳ & Děchtěrenko, 2017, hereafter sparseness). In addition, we added scene typicality (Xiao, Ehinger, Hays, Torralba, & Oliva, 2016) to the comparison. Scene typicality refers to how representative a scene image is for an abstract, semantic category. In that sense, it is not conceptually the same as perceived distinctiveness, which considers how representative an image is with respect to other images in its context. Nevertheless, we expected the two measures to be related, at least to some extent. Indeed, the image context in the current studies consisted of images belonging to the same abstract, semantic category. Therefore, it seemed reasonable to assume that the typicality for that category could be one factor determining how distinctive participants perceived an image to be from the other images in the context. Finally, we also added the perceived distinctiveness measure as a covariate in our analyses of Study 1 and Study 2.

### Methods

#### Participants

In total, 139 people participated in our online distinctiveness study.^3^ Two participants’ data were excluded from the dataset because their demographic data were missing, indicating issues with their account. This brought the total down to 137 (88 women, 48 men, 1 who did not wish to disclose gender). The link to the study was distributed among acquaintances of the researchers and not among undergraduate psychology students, reducing the probability of overlap between the participant pool of Study 3 and those of either Study 1 or Study 2 to a minimum. Ages ranged from 18 to 72 years old (*M* = 33.58, *SD* = 12.69). One hundred one chose to participate in Dutch, 36 chose to participate in English. After choosing their language, all participants gave informed consent by ticking a box. Five gift vouchers were raffled between all participants who completed at least three blocks. The study was approved by the Ethical Committee of the Division of Humanities and Social Sciences, KU Leuven, Belgium.

#### Stimuli, Task and Procedure

When clicking the link to our online study, participants were first asked to choose a language (Dutch or English). Next, some general information about the study was presented and participants provided informed consent by ticking a box. The procedure then moved on to an instruction page and eventually to the online task itself.

**Figure 9:**
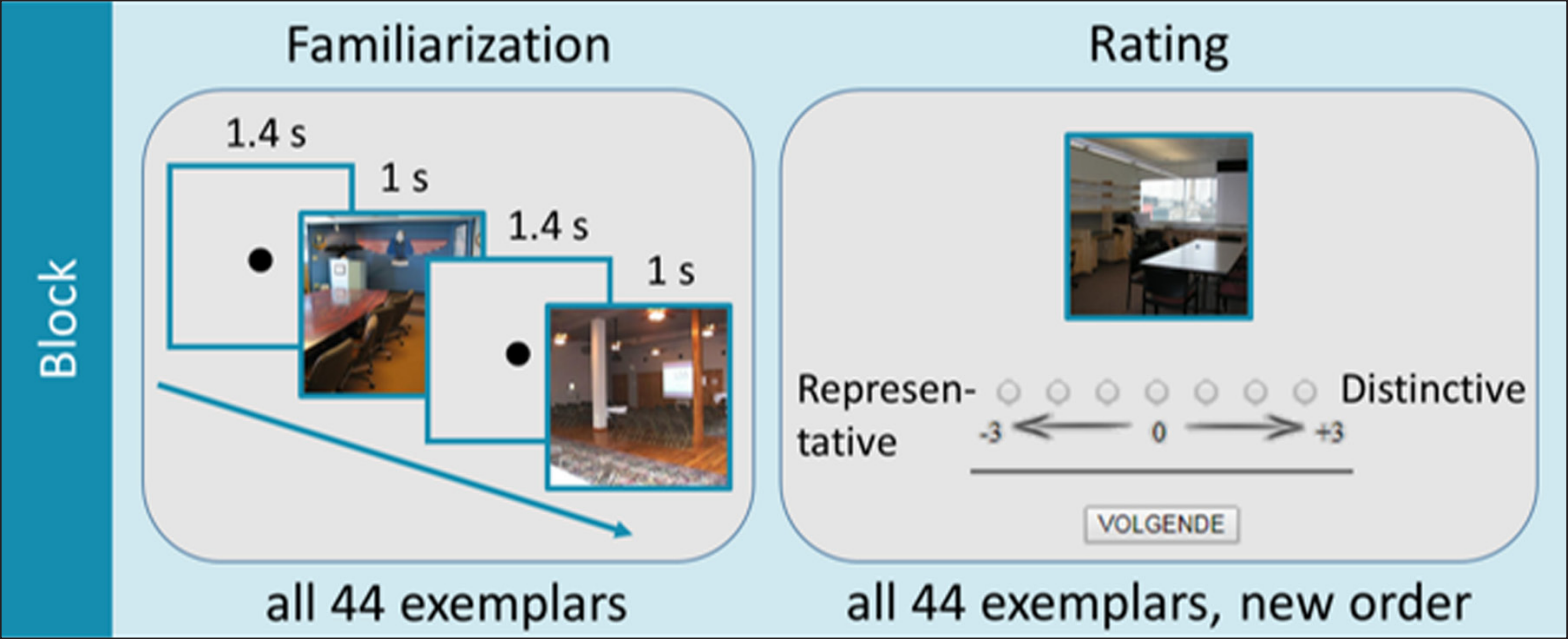
Schematic of one block of the online distinctiveness study.

The task itself was divided into multiple blocks. Each block focused on one of the 14 image categories used in Study 1 and Study 2, and started out with a familiarization phase, followed by a rating phase (see Figure 9). During the familiarization phase, all 44 images of a category were presented on the screen, one by one, in a random order, and with a programmed presentation duration of 1 s, an ISI of 1.4 s, and a size of 512 × 512 px. Note that, due to the online nature of the experiment, actual presentation times and sizes will have differed slightly from those intended. Participants were instructed that these 44 images (per category) functioned as the context with respect to which they had to make their distinctiveness ratings, which was the primary reason for presenting each scene category in a separate block.

Once participants were familiar with the images of the block, they started the rating phase. During this phase, all 44 images were presented again, in a new random order, and participants were asked to rate their distinctiveness with respect to the context of the other images in the category. To do this, they could use a 7-point scale, ranging from –3 (*representative*) to 3 (*distinctive*). A distinctive image was defined as an image that stands out from its context for one or multiple reasons. Furthermore, it was specified that distinctiveness has to do with the extent to which an image distinguishes itself from the other images in its context (e.g., how unusual or unique an image is in a given context). A representative image, on the other hand, was described as one that is largely comparable to the other images and thus fits in really well with the context. In other words, the image is very “normal” given the particular context. Furthermore, participants were informed that they could opt for the middle (0) of the scale in case they found an image neither representative, nor standing out much either. Finally, we emphasized that there were no right and wrong answers and that two images could be equally distinctive, but for entirely different reasons. All of these instructions were provided on the instruction page at the beginning of the study.

Participants could complete as many blocks as they pleased, with a maximum of 14 (since there were no more than 14 categories). They were also given the opportunity to interrupt and resume their participation at a later time. In case a participant left the study in the middle of a block, they would automatically move on to the next block when they logged back in at a later time. Hence, they could not submit additional ratings for the interrupted block. This was to avoid that wearing off of the familiarization would have an influence on the data. However, the ratings submitted up until the point of interruption were still included in the data analysis. Finally, the order of the blocks was randomized across participants.

#### Operationalizations of Distinctiveness

##### Perceived distinctiveness

After collecting distinctiveness data through our online study, each image was assigned a distinctiveness score computed as the mean rating across participants. On average, an image’s distinctiveness score was based on 44 ratings.

##### Sparseness

As mentioned in the Study 3 Introduction, Lukavskỳ and Děchtěrenko’s (2017) sparseness measure reflects how similar an image is to other images its context based on its representation by a CNN pretrained to perform a semantic classification task. In order to compute this measure for the current set of images, we used the CNN-representations of Places-CNN (layer fc7; Zhou, Lapedriza, Xiao, Torralba, & Oliva, 2014), which were already made available by Bylinskii et al. (2015). For each of the 14 categories, we computed all pairwise Euclidean distances between the CNN-representations of their exemplar images. Each image was then assigned a sparseness score that equaled the distance to the third nearest neighbor within its category.

##### CNN-likelihood

As introduced above, Bylinskii et al.’s (2015) contextual distinctiveness measure reflects how likely an image is under the distribution of the images in its context, where images are again represented as CNN-features. We will refer to this measure as CNN-likelihood to avoid confusion with the current distinctiveness measure. To compute the CNN-likelihood scores, we used the code and the CNN-representations (Places-CNN, layer fc7; Zhou et al., 2014) provided by Bylinskii et al. (2015). More specifically, the first step in the code was to reduce the dimensionality of the CNN-representations using principle component analysis (PCA). Next, kernel density estimation was applied to estimate a density distribution over the principle components of the CNN-representations of all images in the context. Because our contexts were too small to be able to apply this technique (44 images per category), we were constrained to using the larger category contexts of Bylinskii et al. (2015), which included the images used here but had a much larger number of category exemplars in total (i.e., a couple of hundreds). Finally, each image used here was assigned a CNN-likelihood score that equaled the negative log likelihood under the estimated density distribution.

##### Typicality

Typicality scores for the images used here were available from Xiao et al. (2016). They presented Amazon’s Mechanical Turk workers with a category label and definition, and asked them to click the three best and three worst illustrations of the scene category in a random array of 20 exemplar images. Typicality scores were calculated as a function of the number of times an image was chosen as best and worst illustration. Thus, these scores reflect the typicality of an image for an abstract, semantic category, rather than the typicality of an image for a given context of other images.

### Results

#### Characteristics of the Perceived Distinctiveness Measure

##### Descriptive statistics

Table 3 presents descriptive statistics for the distinctiveness score per category. As was the case with the categorizability and the shrinkability scores, there seems to be some variation across categories in terms of the mean distinctiveness score of their respective images, with some categories having more distinctive images, overall, than others. Note, however, that all the means and medians are negative, suggesting that the majority of the images received a score at the *representative* side of the scale.

**Table 3:** Descriptive Statistics for Distinctiveness Scores per Category.

	Kitchen	Conference room	Bathroom	Cockpit	Bedroom	Pasture	Living room	Playground	Airport	House	Mountain	Skyscraper	Amusement park	Bridge

Mean	–1.10	–1.08	–0.96	–0.96	–0.93	–0.93	–0.87	–0.87	–0.79	–0.78	–0.78	–0.72	–0.69	–0.67
Median	–1.15	–1.22	–1.18	–1.05	–1.01	–1.18	–1.06	–1.09	–0.99	–1.13	–1.01	–0.80	–0.75	–0.62
SD	0.69	0.83	0.94	0.94	0.82	1.00	0.85	0.90	0.71	1.09	0.84	0.74	0.89	0.67
Min	–2.09	–2.24	–2.24	–2.17	–2.12	–2.36	–2.05	–2.05	–1.93	–2.24	–1.84	–1.81	–2.00	–1.96
Max	1.95	1.22	1.20	2.05	0.86	1.13	1.20	1.28	1.09	1.91	1.23	1.19	1.26	0.88

##### Consistency across participants

**Figure 10:**
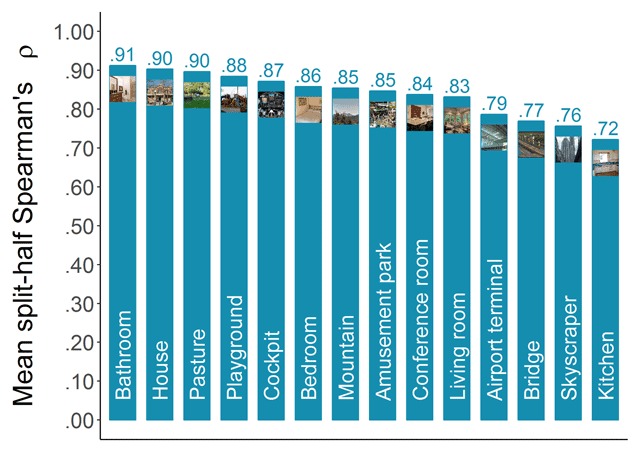
Consistency of distinctiveness scores across participants. Mean-split-half Spearman’s rank correlations were calculated based on 1000 random splits.

To assess the consistency of the distinctiveness scores across participants, we applied the same method as for the categorizability and shrinkability scores (see above and see also Bylinskii et al., 2015). Figure 10 presents the resulting mean split-half Spearman’s rank correlations in function of category. We found high levels of consistency for all categories (ρ’s ranging from .72 up to .91), indicating that participants largely agreed on which images they found distinctive and which they found more representative, and that we obtained a reliable measure of perceived distinctiveness.

##### Comparison with different operationalizations of distinctiveness

**Figure 11:**
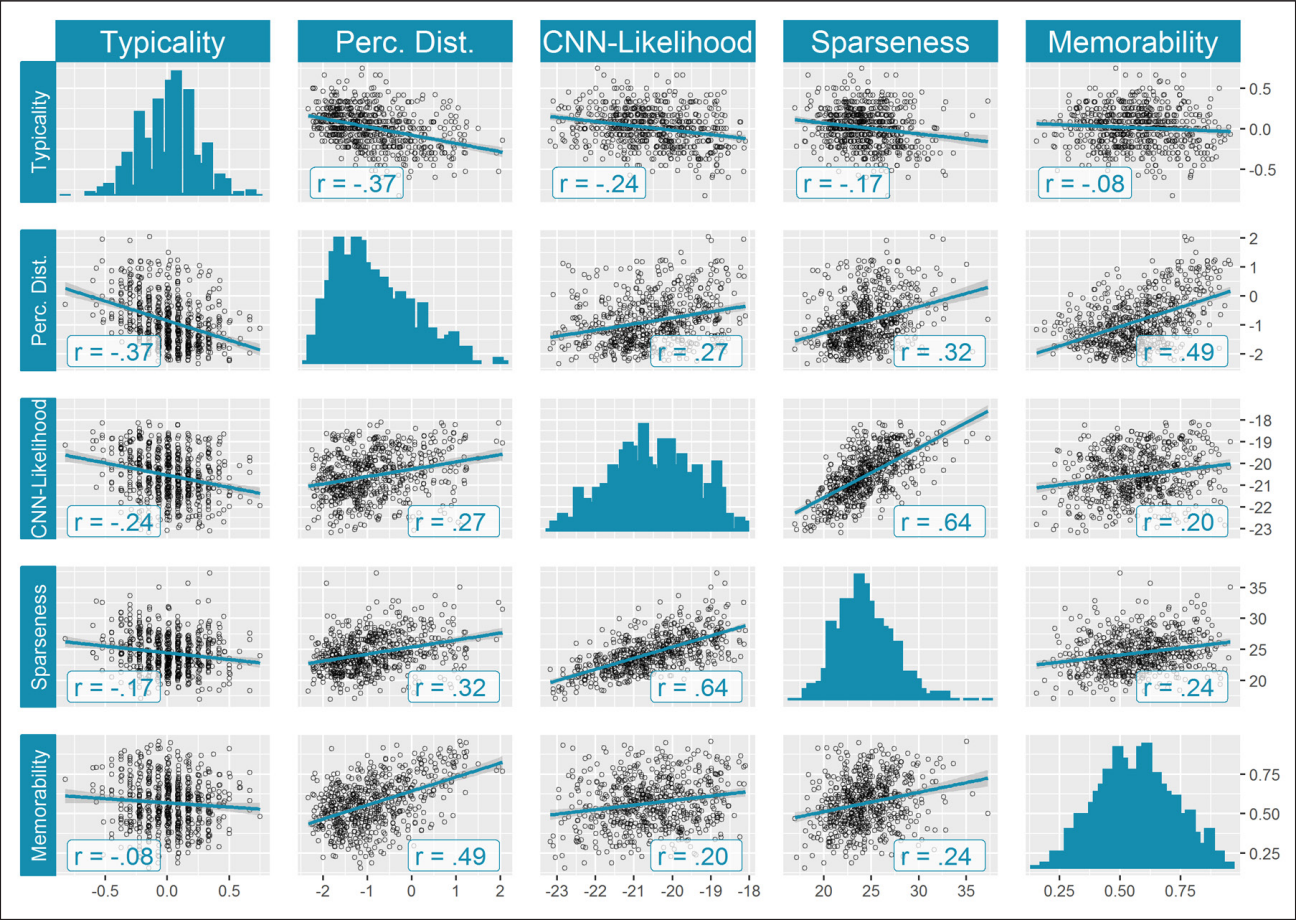
Comparison of different operationalizations of distinctiveness. All reported Pearson correlations except one were significant at an alpha level of .001, even after correcting for multiple testing using the Bonferroni correction. The exception is the correlation between typicality and memorability, for which we found a *p* value of .05.

The pairwise Pearson correlations between all distinctiveness measures are visualized in the top left 4 × 4 submatrix of the correlation matrix presented in Figure 11. A first important observation is that they are all statistically significant and in the expected directions. Notably, the strongest correlation was observed between sparseness and CNN-likelihood: *r*(614) = .64, *p* < .001, which are based on the same, automatically extracted CNN-features. The correlation between each of these CNN-based measures and our behavioral measure is less strong: *r*(614) = .32, *p* < .001 for sparseness, and *r*(614) = .27, *p* < .001 for CNN-likelihood. Note that the context taken into account for the sparseness calculations was the same as for our behavioral measures, whereas it was not entirely the same for CNN-likelihood (see Methods). This could explain why sparseness seems to correlate more strongly with our behavioral measure, although we did not formally test this difference. Furthermore, we also observed the expected negative correlation between our perceived distinctiveness measure and the other behavioral measure, which is typicality: *r*(614) = *–*.37, *p* < .001. Taken together, these results indicate that the current measure of perceived distinctiveness has some important variability in common with other relevant measures from the literature. Simultaneously, it also highlights the considerable proportion of unique variability in our measure of image distinctiveness. We now turn to a reanalysis of Studies 1 and 2, considering the relationship between categorizability/shrinkability and memorability, taking distinctiveness into account.

#### Reanalysis of Study 1 and Study 2

For Study 1, we evaluated our post-hoc explanation of the observed null-relation between categorizability and memorability, which we attributed to a potential masking role of perceived distinctiveness. For Study 2, we wanted to rule out perceived distinctiveness as a potential confounding variable in the observed relation between thumbnail search times and memorability. Critically, both goals of this reanalysis rely on two assumptions: (1) perceived distinctiveness is positively related to memorability, (2) perceived distinctiveness is related negatively with categorizability and positively with shrinkability (and thus negatively with the operationalization in terms of thumbnail search times).

##### Distinctiveness versus memorability

The perceived distinctiveness scores correlated positively with memorability, as predicted: *r*(614) = .49, *p* < .001 (one-sided). It is also interesting to note that out of all operationalizations of distinctiveness compared in Figure 11, our measure showed the highest correlation with memorability (see last row or column in the correlation matrix presented in Figure 11). Apparently, at least some of the variability unique to the current measure is relevant for memorability. To be more consistent with the analyses conducted in Study 1 and Study 2, we additionally computed the correlation between the *z* scores of the perceived distinctiveness scores and the *z* scores of the memorability scores (*z* scores computed per category, as before). This yielded very similar results: *r*(614) = .49, *p* < .001 (one-sided).

##### Distinctiveness versus categorizability and shrinkability

The correlations computed between perceived distinctiveness and categorizability, on the one hand, and perceived distinctiveness and shrinkability, on the other hand, also supported our predictions. For perceived distinctiveness and categorizability (both *z* scored per category), we found: *r*(614) = –.31, *p* < .001 (one-sided), indicating that distinctive images tend to be harder to categorize rapidly. For perceived distinctiveness and shrinkability (both *z* scored per category), we found: *r*(614) = –.15, *p* < .001 (one-sided), indicating that distinctive images are indeed easier to find in the thumbnail search task.

##### Categorizability and shrinkability vs. memorability (reanalyzed)

Encouraged by the support we found for the two underlying predictions outlined above, we conducted the actual reanalysis of the categorizability-vs.-memorability and shrinkability-vs.-memorability relations, where we statistically controlled for perceived distinctiveness by computing partial correlations. After statistically controlling for distinctiveness (*z* transformed), we found a weak, but statistically significant partial correlation between categorizability and memorability (both *z* transformed): *r*(613) = .10, *p* = .01 (one-sided). Note the sign reversal compared to the results of the initial analysis in Study 1. This was predicted by our post-hoc explanation. The new result suggests that, if images are equally distinctive, the memorable ones will tend to be more categorizable. Next, we conducted the same reanalysis for shrinkability and memorability (*z* transformed) and still found a statistically significant partial negative correlation, though smaller than the raw correlation reported in Study 2: *r*(613) = –.18, *p* < .001 (one-sided). Thus, even if all images had equal perceived distinctiveness scores, the more memorable images would still tend to be located faster in the thumbnail search task.

## General Discussion

In the current work, we wanted to contribute to the understanding of image memorability and hypothesized that it (at least partly) depends on goodness of image organization. To evaluate this hypothesis, we exploited two characteristics that have been associated with good organizations: (1) fast, efficient processing, and (2) robustness against transformation. In Study 1, we operationalized fast, efficient processing as an image’s categorizability (i.e., the extent to which an image can be categorized accurately in a rapid-scene categorization task) and tested whether memorable images tend to be more categorizable, as the goodness hypothesis would predict. The results of Study 1 failed to offer support for this prediction. If anything, the nominal correlation was negative. However, we offered a post-hoc explanation arguing that the positive correlation between memorability and categorizability could have been masked by image distinctiveness. To assess this explanation, we collected perceived distinctiveness scores in Study 3 and reanalyzed the relation between memorability and categorizability, this time statistically controlling for distinctiveness. As predicted, the sign of the nominal correlation changed and we found a small but significant positive correlation between memorability and categorizability. Thus, given equal distinctiveness, memorable images do tend to be more categorizable, although the effect is rather small. This finding is consistent with Broers, Potter, and Nieuwenstein (2017), who tested the effect of image memorability on target recognition in an ultra-rapid serial visual presentation sequence. Recognition accuracy was better for memorable targets for all tested presentation durations (varying between 13 ms and 360 ms). The authors argued, much like we did here, that long-term memorability of an image is associated with initial perceptibility and that an image that is hard to grasp quickly is hard to remember later. In Study 2, we tested whether memorable images are more robust against a shrinking transformation. Shrinkability was operationalized using a thumbnail search task, in which shorter reaction times reflect higher shrinkability. The results offered support for the prediction. In order to rule out that this effect was driven by memorable images standing out more from the distractors (i.e., being more distinctive) rather than them being more robust against the shrinking transformation, we reanalyzed the data in Study 3. We statistically controlled for distinctiveness and still found a significant correlation between memorability and shrinkability. Given equal distinctiveness, memorable images still tend to be located faster in the thumbnail search task. Together, these findings offer support for the main hypothesis that goodness of image organization contributes (at least partly) to image memorability. In what follows, we will go more deeply into the results of each individual study.

*Study 1* investigated interstimulus variability in a rapid-scene categorization task. Each image was assigned a categorizability score based on the proportion of participants who correctly categorized the image on congruent trials. We observed a wide range of categorizability scores across the 14 categories, suggesting that some categories might have been easier than others overall. Interestingly, there were also considerable differences within categories. While for some exemplar images, the category information appeared readily accessible after the ultra-short presentation duration of 33 ms, other images were categorized correctly only by a small proportion of participants. These differences were found to be consistent across participants. Indeed, most categories showed high split-half Spearman rank correlations, although there were a few exceptions that scored lower. We attributed this to a restriction of range in the easiest categories and provided analyses supporting that explanation. The high levels of consistency suggest that categorizability could be a meaningful image property too. To the best of our knowledge, no other studies have previously assessed interstimulus variability in rapid-scene categorization systematically, at the image level, as we did here, although some studies have reported differences between broader stimulus categories. For example, previous research indicates that images which are primarily defined by low spatial frequency (LSF) information should be easier to categorize compared to those primarily defined by high spatial frequency (HSF) information (Bar, 2004; Greene & Oliva, 2009; Hochstein & Ahissar, 2002). In particular, it is argued that visual input goes through a fast feedforward sweep of processing to get a first rapid awareness of the conceptual gist of the scene (e.g., fast processing of the, coarse or global, LSF information in the scenes). This rapid implicit perception provides a minimal set of possible input interpretations as an ‘initial guess’ of the visual input. Following this first gist interpretation, feedback connections focus attention to specific low-level elements in the display (e.g., slower processing of the fine, local, HSF information in the scenes). Furthermore, Fei-Fei et al. (2007) found that to achieve the same level of accuracy in describing a perceived scene, shorter presentation durations sufficed for outdoor scenes than for indoor scenes. Notably, the top seven categories scoring highest on mean categorizability in the current study, were all outdoor, except for *cockpit*. The bottom seven were indoor, except for *bridge*. While the current study was primarily aimed at gaining more insight in image memorability and its relation to other variables, we believe that the concept of categorizability is interesting in its own right, which can and should be explored further in future research. Akin to studies on memorability, one question worth investigating is what the underlying factors might be. Here, we have argued that high categorizability reflects fast and efficient processing, which in turn is associated with goodness of organization (as reviewed in the Introduction). However, we do not wish to claim that there could not be additional factors driving categorizability. In fact, Study 3 found that there is also a significant relation between distinctiveness and categorizability, with distinctive images being harder to categorize rapidly. Finally, the current finding might be important for researchers aiming to compare different, non-stimulus related conditions adopting a rapid-scene categorization tasks. They may want to carefully control their stimulus sets in terms of categorizability.

*Study 2* quantified the same set of images on shrinkability. Similar to the categorizability scores, the shrinkability scores also showed considerable variation across the 14 categories. Although we did not formally test this, some categories overall seemed to be located faster in the thumbnail search task than others. Here too, there were considerable differences within categories and those differences were found to be highly consistent across participants. This suggests that shrinkability, like categorizability and memorability, can also be considered an intrinsic image property. In the Introduction, we have argued that this property is driven by the goodness of an image’s organization, with better organizations being more robust against a shrinking transformation. A possible concern, however, is that the operationalization of shrinkability, in terms of reaction times in the thumbnails search task, might reflect more than merely the extent to which an image survives shrinking. As argued in the Interim Discussion, it could be that the reaction times are driven by image distinctiveness. In Study 3, we indeed found a significant negative correlation between distinctiveness scores and shrinkability scores (i.e., distinctive images were located faster), although the effect was small and certainly did not fully explain the variability in the shrinkability scores, nor did it explain away the observed correlation between memorability and shrinkability. In this regard, it is interesting to note that Bainbridge (2017) investigated whether memorable images are more attention-grabbing, which could also result in faster search times in our task. However, in a series of multiple experiments adopting different paradigms, she found evidence that memorability effects cannot be explained away by attention-related phenomena. Based on her results, she argues that memorability is instead a separate image property.

In *Study 3* we quantified the images on a third variable: perceived distinctiveness. While automatically extractable measures for the distinctiveness of scene images were available (Bylinskii et al., 2015, referred to as CNN-likelihood here; Lukavskỳ & Děchtěrenko, 2017, referred to as sparseness here), it remains unclear to what extent they capture what is perceived as distinctive by humans. Therefore, we chose to adopt an approach commonly used in the face distinctiveness literature and had participants rate the distinctiveness in an online study. The resulting perceived distinctiveness scores showed high levels of consistency across participants, indicating that a reliable measure can be obtained using this approach. In addition, we observed that the mean and median perceived distinctiveness score was negative for all categories, indicating that the majority of the images received a score at the representative side of the scale. In fact, this is not too surprising when you consider that a distinctive image was described as standing out from others, unusual or unique. From this, it naturally follows that only a minority of the images would receive very high distinctiveness ratings. Informative regarding the validity of the perceived distinctiveness measure is the explorative comparison with three related variables: (1) CNN-likelihood, (2) sparseness, and (3) typicality for the abstract, semantic category, offered in the Results section. We found that perceived distinctiveness was significantly related to all three variables in the predicted directions (i.e., positively with CNN-likelihood and sparseness, negatively with typicality), although not particularly strongly. This result suggests that there is a considerable proportion of unique variability in our measure of image distinctiveness, which is not captured by the others. At least some of this unique variability seems relevant for memory, as out of all four variables, the highest nominal value for the correlation with memorability was observed for perceived distinctiveness (*r* = .49). Note, however, that these comparison were explorative in nature and that we did not formally test for differences between correlations. Moreover, this is one of the highest values (if not the highest) observed for the correlation between an single factor and scene memorability to date. Finally, perceived distinctiveness also correlated with categorizability and shrinkability in the perceived directions, as discussed above. Taken together, we believe that the current measure of distinctiveness for scene images is a valuable addition to the scene memory literature and contributes to understanding of the concept of distinctiveness and its role in memory.

As a concluding remark, we end on a critical note regarding the theoretical concepts based on which we have interpreted and presented the results. In the line of previous work, we have continuously put forward memorability as an intrinsic, separate image property. In doing so, we mainly wished to emphasize the consistency of the interstimulus variability across participants, contexts, time intervals, etc., as well as the observation that it is not simply synonymous to a single other property, like popularity, interestingness, the ability to capture attention, etc. That is not say that memorability is entirely non-reducible. Rather, we view memorability as a multi-faceted image property, with a variety of underlying factors. The individual factors, as we have hypothesized, are not unique to memorability and some also predict categorizability and shrinkability. In support of that, the current results show that there is a relation between how well images are remembered and how accurately they can be categorized rapidly or how fast they can be found in an array of small images (all whilst controlling for the distinctiveness of the image). As outlined in the introduction, we argue that those shared factors represent what Gestalt psychologists have referred to as goodness of image organization. However, we do believe that translating that directly into extractable image features (i.e., making the shared factors explicit) is still a challenging open question. Because of that, one might rightfully wonder what introducing the concept of goodness contributes and whether the results of the current studies are not better described by focusing purely on the operationalizations (accuracy in the rapid-scene categorization task and reaction time in the thumbnail search task). In addition to the fact that we explicitly designed our studies with this theoretical framework in mind, we argue that it is also useful to explicitly call categorizability and shrinkability measures of goodness, because a certain level of theoretical abstraction can provide a helpful scaffold to think about how interstimulus variability can be shared (partly) across tasks and how we can understand this relation. We believe that the theoretical framework helps the literature to search for factors underlying memorability and goodness alike, rather than documenting which images are remembered better or intuitively appear better organized. However, we do admit that the framework itself can still be improved in terms of its conceptual refinement and possible operationalizations. We regard this as one of the remaining challenges for future research.

In conclusion, the current work considered image memorability as a multi-facet intrinsic image property, with underlying factors at all levels of the visual hierarchy. While previous work has mostly focused on either high- or low-level factors, the contribution of the current work was to focus on a more intermediate level of the visual hierarchy, hypothesizing that part the variability in image memorability resides in the “goodness” of image organization. Approaching this hypothesis from two different angles (i.e., fast, efficient processing in Study 1 and robustness against transformation in Study 2), we found support for this hypothesis, albeit after controlling for distinctiveness (Study 3). However, how goodness translates to directly extractable image features, is still a challenging open question. Finally, it is clear that in addition to factors truly intrinsic to the image, such as goodness, the context plays a role too, as emphasized in previous work (e.g., Bylinskii et al., 2015) and evidenced by the high correlation between perceived distinctiveness and memorability observed here. Whether an image is memorable to people in daily life, will almost certainly depend on a combination of all of those facets.

## Data Accessibility Statement

The novel data discussed in this manuscript can be downloaded from our repository on the Open Science Framework (OSF): https://doi.org/10.17605/OSF.IO/APKSE. The repository contains a folder called “data”, in which one can find three data files in CSV-format, one for each study. In addition, it also contains a README file, providing further clarification on the contents of the folder and how to read it. Finally, there is a folder called “stimuli”, which contains a CSV-file providing a list of the IDs of the selected images, together with the category label (both original and translated), as well as a README file.

## Footnotes

We aimed for 160 participants, which translates to 80 responses per image (on average). This number was chosen based on previous memorability studies (Bylinskii et al., 2015; Isola et al., 2014). Due to organizational reasons (e.g., students signing up but not showing up), the eventual number of participants was 147 instead.

Here, the number of participants was simply determined by how many of the students who had not previously participated in Study 1, were willing to participate in Study 2. Despite the resulting number of responses per image being lower than in Study 1, the consistency of the collected shrinkability scores per image was still very high (see further). Also note that the number of data points going into the crucial analysis (i.e., the correlation between memorability and shrinkability) is the number of images (616), which was the same as in Study 1.

In Study 3, the number of participants was determined by how many acquaintances we could convince to participate within a timeframe of 2 months.
